# Do Age and Co-morbidy, Among other Factors, affect Length of Hospital Stay following Total Knee Arthroplasty

**DOI:** 10.5704/MOJ.1807.005

**Published:** 2018-07

**Authors:** IS Komang-Agung, O Sindrawati, PS William

**Affiliations:** Department of Orthopaedics, Airlangga University, Surabaya, Indonesia; ^*^Department of Pathology, Widya Mandala Katholic University, Surabaya, Indonesia; ^**^Department of Orthopaedics, Surabaya Orthopedics and Traumatology Hospital, Surabaya, Indonesia

**Keywords:** length of stay, TKA, osteoarthritis knee

## Abstract

**Introduction:** The only treatment for grade IV knee osteoarthritis is total knee arthroplasty (TKA) irrespective of the age of the patient. Most of the grade IV OA patient are elderly and most likely to have some comorbidities. Age and comorbidities are the major reasons for patient’s reluctance to undergo TKA. A clinical pathway with standard length of stay (LOS) could justify the patient’s hesitation for TKA. The aim of this study was to determine the factors, including age and comorbidity, that affect the LOS of patients treated with TKA. **Materials and Methods:** This is a retrospective study of TKA patients in Surabaya Orthopedics and Traumatology Hospital from January 2011 to July 2017. Preoperative comorbidities were scored using Charlson Comorbidity Index (CCI) and physical status by ASA (American Society of Anesthesiologist), classification for age, sex, BMI, blood loss, operation time, method of anaesthesia and postoperative day of rehabilitation were recorded as factors potentially affecting LOS. The discharge criteria for the patients were ability to ambulate to the bathroom and clean operative wound with no complications. The data obtained were analysed statistically.

**Results:** The average LOS was 5.58 days, ranging from three to eight days. There were no demographic factors that affected the patients’ LOS. BMI, ASA, CCI, and blood loss did not significantly affect LOS. Operation time was between 90-140 minutes, and spinal anaesthesia showed significant longer LOS, but within the average.

**Conclusion:** Age and comorbidity did not affect length of stay in TKA patients.

## Introduction

Since 2000 to 2016, global life expectancy had risen by five years from 72 to 77.5 years^1^. However, the rise in life expectancy was followed by increase in degenerative diseases such as osteoarthritis. In the United States alone, the increase in longevity and body mass index (BMI) had doubled the prevalence of knee osteoarthritis since mid-twentieth century^2^. Throughout the world, osteoarthritis of the knee and hip is the eleventh high contributor to disability^3^. In Southeast Asia, the prevalence of osteoarthritis is as high as 62% in Brunei and 40-42% in Malaysia and Thailand. Indonesia has similar demographic features to Malaysia and Thailand, and socio-cultural similarity as well^4^.

Total joint arthroplasty is an effective and economical procedure to treat knee and hip osteoarthritis^5^. The mortality of the procedure has decreased to 0.3% between 2007-2010 in the United States^6^. However, it still poses some common complications such as pneumonia, pulmonary embolism and infection^7^. Moreover, older patients are more likely to have longer length of stay (LOS) and more complications. Geriatrics comprises the largest proportion of grade IV knee OA patients. Hesitation of the procedure is still common, due to the fear of possible complications. There is also a common cultural belief among our population that geriatrics should avoid surgery due to their assumed frailty^8^.

One of the indicators of good quality care is the ability to shorten patient’s length of stay^9^. Shortening the LOS is one of the ways to reduce the cost burden of the procedure but the shortening of LOS should not exceed the goal of patient’s well-being on the day of discharge. In western countries where nursing home and similar institutions are easy to access, short LOS do not always end up at home. Patients are discharged to be taken care in the nursing home^10^.

The aim of this study is to analyse factors that affect LOS of the TKA patients.

## Materials And Methods

This is a retrospective cross-sectional study of primary TKA patients from Surabaya Orthopedics and Traumatology Hospital during the period 2011-2017, reviewed and approved by the Internal Institutional Review Board. The procedures for management of TKA have been laid out by the hospital, and involve several orthopaedic surgeons, anaesthesiologists, and rehabilitation specialists. Patients who had undergone revision surgery and neglected TKA cases were excluded from the study. Demographic data such as, age, sex, ethnic, body mass index (BMI), laboratory findings, radiological findings were recorded. Preoperative comorbidities were scored using Charlson Comorbidity Index (CCI), and physical status according to American Society of Anesthesiologist (ASA). The CCI was calculated by comorbidities and ASA assessed by the anaesthesiologist preoperatively. All data were extracted from medical records. The patients’ Charlson Comorbidity Index were grouped as CCI ≤3 or CCI>3. BMI of <18.5 was classified as underweight, 18.5-24.9 as normal; 25-29.9 as overweight, 30-34.9 as Obese class I, 35-39.9 as Obese class II and ≥40 as Obese class III^11^.

The standard LOS according to our hospital clinical pathway is six days, allowing patients to fulfill the post-operative rehabilitation program. The rehabilitation begins on the first post-surgery day. The first day of rehabilitation program includes quadriceps isometric and isotonic exercises. The patient should be able to stand on the third day after operation and continue to partial weight bearing walking with the aid of walker on the fourth day. The discharge criteria include the patient’s ability to ambulate to the bathroom, clean operative wound and no surgical complications (infection, dislocation, and pain). The patients are told to keep the wound dry and to return in one week for wound inspection and evaluation.

Data was analysed statistically using the Mann-Whitney U test for comparison within variable with two categories and Kruskal-Wallis test for comparison of variables with three or more categories.

## Results

There were 158 TKA patients, 31 male and 127 female (80.4%). The majority of cases were within the age of 60-69 (44.3%). The dominant patient’s BMI was normal (33%) and overweight (37.3%) but gender, age, race, CCI, ASA, and BMI did not show significant difference in LOS. The average LOS of all patients was 5.58 days and the median was six days. Patients’ demographic factors and intraoperative factors are presented in Table I.

**Table I:** Demographic and preoperative data of the patients

Factors	N (%)	LOS	p-Value
Gender			
Male	31 (19.6)	5.45	0.489^Ɨ^
Female	127 (80.4)	5.61	
Age			
<50	2 (1.3)	6.50	0.228^*^
50-59	31 (19.6)	5.48	
60-69	70 (44.3)	5.44	
70-79	44 (27.8)	5.66	
≥80	11 (7.0)	6.18	
Race			
Malay	37 (23.4)	5.55	0.507^Ɨ^
Chinese	121 (76.6)	5.68	
Indonesian			
BMI			
<18.5	8	4.88	0.335^*^
18.5-24.9	52	5.83	
25-29.9	59	5.54	
30-34.9	20	5.45	
35-39.9	10	5.60	
≥40	4	5.00	
CCI			
≤3	122 (77.2)	5.52	0.280 ^Ɨ^
>3	36 (22.8)	5.75	
ASA PS			
1	5 (3.2)	5.40	0.740^*^
2	105 (66.5)	5.57	
3	48 (30.4)	5.60	
Intraoperative Factors			
Type of Anaesthetics			
Spinal Anaesthesia	136 (86.1)	5.68	0.003 ^Ɨ^
General Anaesthesia	22 (13.9)	4.91	
Operating Time			
<90	35 (22.2)	6.00	0.004^*^
90-99	47 (29.7)	5.81	
100-109	12 (7.6)	5.67	
110-119	4 (2.5)	5.25	
120-129	35 (22.2)	5.00	
130-139	3 (1.9)	5.67	
≥140	22 (13.9)	5.32	
Blood Loss			
<100	7 (4.4)	5.14	0.083^*^
100-199	123 (77.8)	5.71	
200-299	17 (10.8)	5.06	
300-399	8 (5.1)	5.25	
400-499	1 (0.6)	4.00	
≥500	2 (1.3)	5.50	

BMI= Body Mass Index; CCI= Charlson Comorbidity Index; ASA PS= American Society of Anesthesiologist Physical Status; LOS= length of stay

ƗMann-Whitney U test

* Kruskal-Wallis test

Factors statistically found to contribute to patients’ LOS are type of anaesthesia and duration of the operation. The longer LOS was observed in patients anaesthetised with spinal anaesthesia (5.68 days), compared to the general anaesthesia group (4.91 days) (p=0.003). Although shorter operating time (<90 minutes) was recorded in 22.2% cases, it significantly correlated with longer LOS (6 days), while the shortest LOS (5 days) was conducted in 120-129 minutes (p=0.004). The average intra-operative blood loss was 160.24 ml. This did not significantly affect the LOS (p=0.083). Whether the patients began rehabilitation on Day 1 or Day 2 was not statistically significant (p=0.129) (Table II).

**Table II:** Day the patients began rehabilitation post-operative (Day 0 operation day)

Day of Starting Rehabilitation	N (%)	p-Value
1	130 (82.3)	0.129^Ɨ^
2	28 (17.7)	

ƗMann-Whitney U test

## Discussion

There were no demographic factors which significantly affected patients’ LOS. There is no difference in the average LOS amongst all age groups. This finding was contrary with many previous studies^8,12^. Fang *et al* in their study defined the LOS as discharge from hospital to home or other nursing home. The LOS was significantly different in patients above age of 80 years (3.98 days) but most of those patient were discharged to nursing home or others (63%). Of significance was the age of patients admitted to ICU which was 71 to 80 or beyond^8^. All 158 TKA patients in our study did not require admission to ICU after surgery. This may signify that elderly patients do not always have worse outcome following knee arthroplasty. In our institution patients were discharged to home and family, after ensuring that they were independently mobile for toilet and personal hygiene and was the objective of early rehabilitation as one of the goals and indicator of successful TKA treatment. There was one patient who stayed for three days and ten patients who stayed for eight days due to non-medical reason. The reasons included domestic reasons (absence of care giver), the cultural belief that hospital discharge should not be on Saturday or Sunday, and patient’s preference to continue rehabilitation programme in the hospital rather than at home.

The patients’ BMI also did not affect patient’s LOS. This result was similar to a large cohort study in which BMI status had no impact on patients’ LOS following elective TKR^14^. However, other studies, one conducted in UK and the other in USA found that higher BMI was associated with longer LOS^15,16^. Meanwhile, Walters *et al* stated in their study that preoperative nutritional assessment and optimisation of patients’ status prior to surgery may reduce the overall LOS^17^. There was no difference in LOS between different ethnicities. Previous studies found that Malays generally had better functional improvements postoperatively compared to Chinese. Currently there is limited evidence of studies comparing LOS between Malay and Chinese ethnicity^18^.

Comorbidity, assessed using CCI or ASA, was not correlated with increasing LOS. This finding is supported by other studies^15,20^. Some studies have found positive correlation between comorbidities and LOS, but there is still limited evidence^21^. Careful pre-operative assessment may explain why comorbidity did not affect patients’ LOS. In the geriatric population, preoperative assessment may reduce surgery delay LOS by 31-45%, and medical complications^22^.

Contrary to many previous studies, patients anaesthetised with spinal anaesthesia in this study generally had longer LOS than those who underwent general anaesthesia. Spinal anaesthesia is known for its advantages, such as its potential to reduce bleeding and incidence of thromboembolic events. However, spinal anaesthesia is also known to cause some delay of motor recovery to the lower limb, thus hindering early post-operative mobilisation^23^. This could explain why patients in the spinal anaesthesia group were discharged later than those in the general anaesthesia group. Other studies also showed that type of anaesthetics used generally did not affect patients’ LOS^24^. Furthermore, a recent review concluded that general and regional anaesthesia are equally effective in joint arthroplasty, in respect of post-operative outcome^25^.

Interestingly, patients with shorter operative time showed longer LOS. This was contradictory to previous findings which found increased operative time generally led to longer LOS and more overall complications^26,27^. However, there was also another study which found that operative time was not an important predictor of LOS after hip arthroplasty^26^. This negative correlation may be contributed by other factors outside the scope of this study, like performance bias, as different surgeons performed the arthroplasty procedures in our hospital.

Blood loss did not affect patients’ LOS significantly in our study, and a similar study found less blood loss led to less hospital resource use^28^. Even so, our study did not include all aspects of blood loss, like preoperative and postoperative haemoglobin levels to detect any drop after the procedure. These factors should be included in future studies.

There was no difference of LOS between patients who began their rehabilitation between 1-4 postoperative days. However, there were only four patients who began their rehabilitation after day two, and one of the patients who began rehabilitation on Day 4 also had the longest LOS of all patients. Raut *et al* found that the starting day of the rehabilitation did not necessarily affect LOS^15^. The majority of patients underwent the standard procedure to begin early rehabilitation on Day 1, with better outcome, similar to previous studies^18,29,30^. Further study may help to assess other aspects of rehabilitation which may affect LOS.

The authors recognise various limitations in the present study as it was retrospective and non-randomised, with the data obtained from a single medical centre. Social factors should be included in future studies as it may influence patients’ LOS. For example, there is a belief among Indonesian Chinese that patients should not be discharged from hospital on Saturday, and also other factors such as the absence of caregiver at home. Further studies were also needed to assess other aspects pertaining to rehabilitation outcome, namely the day independent ambulation was achieved, or the day full range of motion of the knee was attained Nevertheless, this study offers a unique perspective on joint arthroplasty in our community.

## Conclusion

Length of stay for TKA is not affected significantly by age and comorbidities. This provides new insight that old age and comorbidities are not always associated with worse outcome or longer length of stay.

## Conflict Of Interest

All authors declare no conflict of interest in this study. No specific grant or financial support from any agency were received for the research, authorship and/or publication of this article.
